# The Impact of Burnout on Body Composition in Medical Staff

**DOI:** 10.3390/medicina62040690

**Published:** 2026-04-03

**Authors:** Sabinne-Marie Albișteanu, Adina Carmen Ilie, Anca Iuliana Pîslaru, Ramona Ștefăniu, Ana-Maria Turcu, Gabriela Grigoraș, Iulia-Daniela Lungu, Ioana Dana Alexa

**Affiliations:** Grigore T. Popa University of Medicine and Pharmacy Iasi, 700115 Iași, Romania

**Keywords:** burnout, healthcare staff, body composition, bioimpedance

## Abstract

*Background and Objectives*: Burnout syndrome is a major challenge among healthcare workers, affecting both mental and physical health. Although stress may influence physiological systems, this study does not directly assess biological mechanisms, and interpretations should remain associative. Stress-related associations may be reflected in body composition, but objective data are limited. This study aims to assess the associations between burnout, body composition, and quality of life in medical staff, using bioimpedance as the main tool. *Material and methods*: The cross-sectional study included 95 medical staff (36 resident doctors, 13 specialists/consultants, 31 nurses, 15 nursing assistants), 75.8% women, mean age 38 ± 10 years, conducted in the medical and surgical departments of Dr. C.I. Parhon Clinical Hospital, Iași, between January and March 2024. Burnout was assessed using the Maslach Burnout Inventory; quality of life was evaluated with SF-12; body composition was measured by bioimpedance (BMI, muscle mass, fat mass, bone mass, hydration, basal metabolism, metabolic age). Statistical analyses included ANOVA, Kruskal–Wallis, and Spearman correlations. *Results*: Resident physicians had higher muscle, bone mass and basal metabolism, with lower adipose tissue compared to other categories (*p* < 0.05). Total burnout and exhaustion were positively correlated with muscle mass (r = 0.247, *p* = 0.016), bone mass (r = 0.219, *p* = 0.033), basal metabolism (r = 0.219, *p* = 0.033) and negatively with QoLM (r = −0.315, *p* = 0.002). Other body variables, including BMI, total adipose tissue, and water level, showed no significant correlations. These associations may be influenced by age, sex, and professional category and do not imply causality. *Conclusions*: Age, profession, and work seniority are associated with burnout and specific changes in body composition. However, these findings are correlational, and bioimpedance-derived parameters do not reflect direct biological stress mechanisms.

## 1. Introduction

Interest in burnout syndrome has grown considerably over the last decade, reflecting the increasing challenges of modern professional environments and the dynamics of occupational stress [1].

Among healthcare staff, the prevalence of burnout varies between 10 and 37% and is influenced by factors such as age, experience, specialty, and coping strategies [2,3,4,5,6].

One of the first elaborate and refined definitions of burnout was provided by Maslach and Leiter, who considered it a delayed reaction to occupational stress, characterized by exhaustion, cynicism, and inefficiency [7]. Later, the theory was substantiated by Hu et al., who identified a determinism between increased resources, dedication, and the avoidance of burnout, who highlighted the protective role of resources and dedication in mitigating burnout, identifying early levers of resilience [8].

At the same time, Schaufeli and colleagues highlighted the occurrence of burnout even in high-performing staff, manifested by profound exhaustion, emotional and cognitive dysfunction, and psychological detachment [9,10,11].

All major collective crises impose a permanent need to consolidate these definitions, dynamically and unpredictably rewriting the expressions of contemporary burnout. Emerging forms of burnout linked to new crises or stressors require further investigation. Research should go beyond traditional psychosomatic and behavioral assessments to examine associations between stress and body composition [7,8,9,10].

Associations between stress and body composition are a subject that has been little explored in the literature. Interest in understanding the physical manifestations of burnout has driven efforts to develop reliable assessment methods for body composition and related parameters [11].

Individual differences in vulnerability to stress can be partly explained by personality traits: neuroticism promotes exhaustion, while extraversion and resilience have a protective effect. Contemporary personality models, such as Cloninger’s PEN model, correlate these traits with physiological functioning, suggesting the existence of physiological patterns susceptible to burnout [12].

At the physiological level, stress activates the sympathetic nervous system and the hypothalamic–pituitary–adrenal (HPA) axis, increasing metabolic and cardiovascular parameters [12]. In 1930, Selye described stress as a general adaptation syndrome, with stages of alarm, resistance, and exhaustion [13]. Later, in 1988, Sterling and Eyer, through the concept of allostasis, explained how repeated adaptation to stress alters physiological set points, potentially leading to hyperexcitability and maladaptive responses [14]. HPA axis dysfunction is central to burnout, involving abnormal cortisol levels and impaired negative feedback. Under stress, CRH stimulates the secretion of ACTH and subsequently cortisol, which mobilizes energy resources but inhibits functions considered non-essential: digestion, sleep, reproduction, and immunity. Chronic exposure to cortisol disrupts these systems, generating the classic symptoms of exhaustion [12].

Both hyperactivity and hypoactivity of the HPA axis can occur in people with burnout, suggesting the existence of distinct pathophysiological subtypes. These mechanisms may contribute to changes in body composition, as repeated activation of the HPA axis promotes increased lipid stores, redistribution of visceral fat, fluid retention, and the development of insulin resistance. Furthermore, the sustained activation of inflammatory markers and endocrine changes can alter the ratio of muscle mass to fat mass, increase protein catabolism, or influence fluid homeostasis. Studies on the “Karoshi” phenomenon in Japan have highlighted associations between emotional exhaustion and altered lipid profiles, suggesting cardiometabolic implications of occupational stress. Other investigations have shown that the release of corticosteroids and catecholamines can induce insulin resistance and promote the onset of type II diabetes, mechanisms relevant to alterations in body composition [15].

In this context, electrical bioimpedance has emerged as a non-invasive, accurate, and widely applicable method for analyzing body composition and monitoring stress-induced changes. With a history of nearly two centuries, bioimpedance techniques have evolved from fundamental observations on tissue conductivity to modern instruments used to estimate total and regional body composition, including fluid compartments, fat mass, and muscle mass [16]. Bioimpedance thus provides an objective window into the physiological variations generated by occupational stress and allows investigation of how burnout is reflected in the body–metabolic parameters of medical staff.

Furthermore, modern, informed, and progressive medicine understands, in the context of the global trend of aging and increased life expectancy, the importance of investigating and ensuring the quality of life of individuals. Ultimately, screening questionnaires that require constant improvement, by reconsidering and identifying new constantly changing stressors, giving due importance to the quality of physical and mental life, and studying stress-related associations, are the first levers for increasing individual and collective resilience to burnout. At the same time, the perspectives opened up by the subject of this research mean awareness of the major risks of this pathology and accountability for screening, diagnosis, prevention, and combating methods in any future crisis situation.

Thus, bioimpedance, used and approved in cardiological explorations, recognized for its non-invasive and accessible profile, represented a new point of originality in this work, by initiating new levers for investigating the associations between burnout and body composition. Determining readily available parameters such as muscle strength, visceral adipose tissue, basal metabolism, water level, bone mass, or metabolic age can be a simple and inexpensive way to monitor the effects of burnout over time. It should be emphasized that these findings are associations with burnout and do not provide evidence of direct biological stress mechanisms. Understanding the interaction between chronic occupational stress, stress-related associations, and compositional changes in the body can contribute to the development of integrated strategies to protect the physical and mental health of healthcare staff.

This study aims to investigate the impact of burnout on the body composition of medical staff using bioimpedance and to assess their quality of life.

## 2. Materials and Methods

### 2.1. Study Design

This study used a cross-sectional observational design, simultaneously assessing the level of burnout, body composition, and quality of life of medical staff.

### 2.2. Study Setting

The study was conducted in two departments, one medical and one surgical, at the Dr. C.I. Parhon Clinical Hospital in Iași, between January and March 2024, during which time participants were recruited, questionnaires were administered, and bioimpedance analysis was performed. A total of 95 healthcare staff (resident doctors, specialists/primary care physicians, nurses, and nursing assistants) were evaluated for associations between burnout and quality of life.

### 2.3. Participants

Inclusion criteria:

Age over 18;

▪ Professional affiliation with the medical field (primary care physician/specialist, resident physician, nurse, nursing assistants);
▪ Completion of informed consent for participation in the study;
▪ Body weight under 150 kg.

Exclusion criteria:

▪ Age under 18;
▪ Occupation outside the medical field;
▪ Refusal to complete the informed consent form;
▪ Body weight over 150 kg.

Participants were recruited consecutively based on these criteria and gave informed consent. Assessments were conducted in a post-critical period, distant from the COVID-19 pandemic, during the cold season.

### 2.4. Response Rate

A total of 95 eligible medical staff were invited to participate, and all completed the study, resulting in a response rate of 100%. No participants withdrew, and no missing data were recorded.

### 2.5. Methods

The methods described below were designed to achieve the study objectives outlined in the introduction.

Participants were assessed using the Maslach Burnout Inventory for stress levels and the SF-12 questionnaire for physical (QoLF) and mental (QoLM) quality of life. Body composition was measured via bioimpedance using the TANITA BC 545N scale. Variables obtained included BMI, segmental and total muscle mass, percentage of subcutaneous and visceral fat, bone mass, water level, muscle strength, metabolic age, and basal metabolism. All variables were fully collected; no missing data were recorded.

### 2.6. Work Stages

1. Establishing inclusion/exclusion criteria and study protocol.
2. Obtaining consent to use Romanian SF-12 questionnaire.
3. Validating the TANITA BC 545N scale (TANITA Corporation, Tokyo, Japan) for scientific use.
4. Completing legal formalities for assessment tools and scales.
5. Designing informed consent forms.
6. Obtaining approval from the Research Ethics Committee of “Grigore T. Popa” University of Medicine and Pharmacy and the Dr. C.I. Parhon Clinical Hospital.
7. Administering questionnaires and conducting bioimpedance analysis while maintaining participant anonymity.

### 2.7. Parameters Studied

The following parameters were assessed to evaluate associations with burnout and quality of life:

1. **Demographic parameters**: Sex, age, occupation, specialization.
2. **Body composition parameters**: BMI, segmental and total muscle mass, percentage of subcutaneous and visceral adipose tissue, bone mass, water level, muscle strength, metabolic age, basal metabolism (kcal)
  ▪ Water level normal values between 45 and 60% in women; <45%—dehydrated; >60%—hyperhydrated; in men, between 50 and 65%—normal values.
  ▪ Bone mass: Normal values depending on sex and weight—female: G < 50 kg = 1.95 kg bone mass; 50 kg < G < 75 kg = 2.40 kg bone mass; G ≥ 75 kg = 2.95 kg bone; male: G < 65 kg =2.65; 65 kg < G < 95 kg = 3.29 kg bone; G > 95 kg = 3.69 kg bone.
  ▪ Adipose tissue in organs: Normal values between 1 and 12; 12–19—moderately increased; >19—severely increased.
  ▪ Metabolic age (the age of the body, estimated using scales, may differ from the chronological age stated on the ID card)—calculated by the bioelectrical impedance analysis (BIA) device software (TANITA BC-545N).
  ▪ Muscle mass: Normal values between 1 and 4—fatty tissue predominates over muscle mass; 5—balance between muscle mass and adipose tissue; 6, 7, 8, 9—muscle mass predominates over fatty tissue.
  ▪ Basal metabolism in Kcalories and Kjoules—what the body consumes at rest, without any activity; the higher the values, the faster the body burns calories (catabolism).
3. **Burnout parameters**: Exhaustion, cynicism, inefficiency and total burnout score using the Maslach Burnout Inventory questionnaire—high level of burnout (Exhaustion ≥ 27, Cynicism ≥ 10, Inefficiency 0–33), medium level of burnout (Exhaustion 19–26, Cynicism 6–9, Inefficiency 34–39), low level of burnout (Exhaustion 0–18 Cynicism 0–5, Inefficiency ≥40) [17].
4. **Quality of life parameters**: SF-12 questionnaire summary scores for physical (QoLF) and mental (QoLM) quality of life

### 2.8. Assessment of Quality of Life

We used a validated Romanian version of the SF-12, distributed in paper format to all participants and completed under supervision. Scores below 50 indicated decreased quality of life, while scores above 50 indicated normal quality of life.

### 2.9. Permission and Validation

Permission to use the SF-12 for scholarly research purposes was confirmed by the original author, Prof. John E. Ware Jr., who also provided the official manual detailing scoring and use. No additional translation or validation was required, as the Romanian version has been previously validated in published studies [18].

### 2.10. Data Sources and Measurement

All assessments were standardized across participants using validated instruments.

### 2.11. Bias Control

Selection bias was minimized through consecutive recruitment and clear criteria. Information bias was reduced by using the same team for all measurements.

### 2.12. Sample Size

The study included all eligible medical staff available during the recruitment period (*n* = 95). No formal statistical sample size calculation or power analysis was performed prior to data collection, as all available participants were included. This approach ensured that the study captured the full population of interest within the selected departments.

### 2.13. Quantitative Variables

Variables were analyzed as continuous or categorized per clinical and scientific guidelines.

### 2.14. Statistical Analysis

Statistical analysis was performed using IBM SPSS Statistics 25 and Microsoft Office Excel/Word 2013. Quantitative variables were tested for normality using the Shapiro–Wilk test and were expressed using means and standard deviations or medians with interquartile ranges.

Normally distributed independent quantitative variables were tested between two independent groups using the Student’s *t*-Test/Welch *t*-Test (depending on the equality of variances between groups observed using the Levene test), while non-parametrically distributed independent quantitative variables were tested between groups using the Mann–Whitney U test.

Normally distributed independent quantitative variables were tested between multiple independent groups using the One-Way ANOVA test (along with Tukey HSD post hoc tests), while non-parametrically distributed independent quantitative variables were tested between groups using the Kruskal–Wallis H test (along with Dunn–Bonferroni post hoc tests).

Correlations between normally distributed variables were tested using Pearson’s correlation coefficient, while correlations between non-parametrically distributed variables were tested using Spearman’s rho correlation coefficient.

Qualitative variables were expressed as absolute frequencies and percentages.

Comparisons between occupational groups were performed using appropriate tests: the Kruskal–Wallis test for non-parametric variables and One-Way ANOVA for normally distributed variables, followed by post hoc tests (Dunn–Bonferroni or Tukey HSD) if the overall test was significant. This approach allows for the highlighting of differences in body composition between professional categories.

### 2.15. Ethical Procedures

All participants provided informed consent. The study was approved by the Research Ethics Committee of the “Grigore T. Popa” University of Medicine and Pharmacy and the Dr. C.I. Parhon Clinical Hospital Romania (protocol code 9891 and date of approval—16 December 2020).

## 3. Results

### 3.1. Response Rate

Of all eligible medical staff invited to participate, 95 completed the study, yielding a 100% response rate. No participants withdrew or had missing data.

### 3.2. Participant Characteristics

Most respondents were resident physicians (36, 37.9%), followed by nurses (31, 32.6%), nursing assistants (15, 15.8%), and specialist/primary care physicians (13, 13.7%). The majority of participants worked in medical departments (75.8%) and were female (75.8%).

The mean age was 39.9 ± 10.3 years (median 38), with 57.9% of participants aged over 35 years (Table 1).

These findings describe the demographic and professional structure of the study population and support the interpretation and generalizability of the results.

### 3.3. Body Composition by Occupation

#### 3.3.1. Body Mass Index (BMI)

The median BMI differed between occupational groups (Kruskal–Wallis H, *p* = 0.048), with a downward trend in BMI values from the nursing occupation to the specialist doctor occupation, though post hoc tests did not reach significance (Table 2).

#### 3.3.2. Muscle Mass

According to our results, resident physicians had significantly higher muscle mass compared to specialist physicians (*p* = 0.038).

#### 3.3.3. Subcutaneous Adipose Tissue

Resident physicians exhibited significantly lower subcutaneous adipose tissue compared to nurses (*p* = 0.006) and nursing assistants (*p* < 0.001).

#### 3.3.4. Water Content

The water content was significantly higher in resident physicians compared to nurses (*p* = 0.001) and nursing assistants (*p* = 0.001).

#### 3.3.5. Bone Mass

Resident physicians showed significantly higher bone mass compared to specialist/primary care physicians (*p* = 0.023).

#### 3.3.6. Visceral Adipose Tissue

Resident physicians had a significantly lower percentage of visceral adipose tissue compared to nurses (*p* = 0.042).

#### 3.3.7. Metabolic Age

The metabolic age was significantly lower in resident physicians compared to nurses (*p* = 0.003) and nursing assistants (*p* = 0.001).

#### 3.3.8. Basal Metabolic Rate

Resident physicians demonstrated a significantly higher basal metabolic rate compared to specialist/primary care physicians (*p* = 0.003).

All body composition, demographic, and occupational variables were available for all participants—there were no missing data for any of the variables of interest.

### 3.4. Burnout and Quality of Life

#### 3.4.1. Inefficiency Score

**Figure 1:**
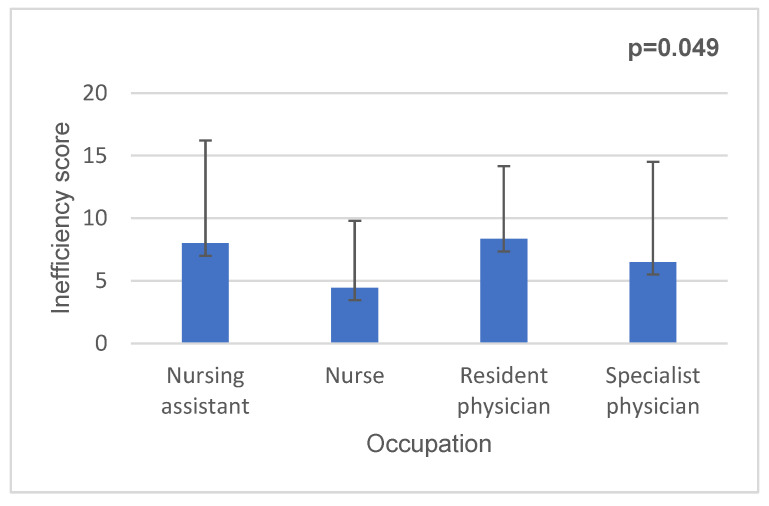
Inefficiency score and occupation in medical staff.

Resident physicians had a significantly higher inefficiency score compared to nurses (*p* = 0.049) (Figure 1).

#### 3.4.2. Mental Quality of Life (QoLM)

**Figure 2:**
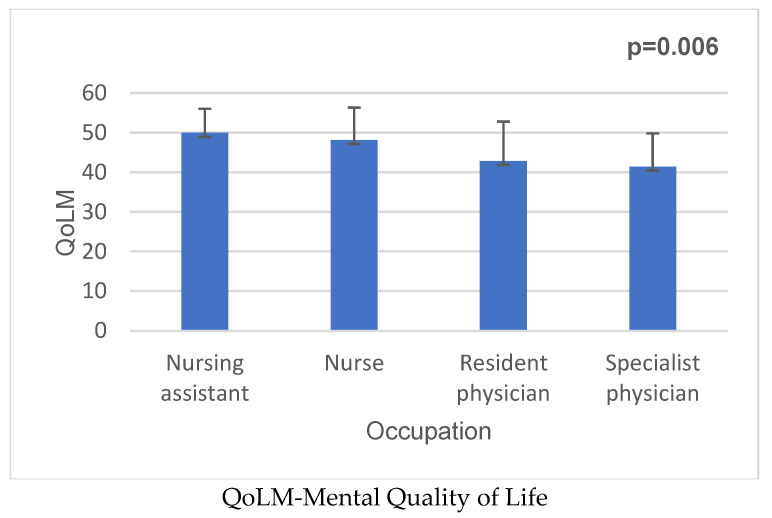
QoLM and occupation in medical staff.

Resident physicians reported significantly lower mental quality of life compared to nurses (*p* = 0.006) (Figure 2).

#### 3.4.3. Other Burnout Dimensions (Exhaustion, Cynicism, Total Burnout) and Physical Quality of Life (QoLF)

For exhaustion, cynicism, burnout, and QoLF scores, no significant differences were observed between the occupational groups (*p* ≥ 0.05; descriptive details are presented in Table 3). These negative results are included for transparency and allow the reader to fully assess the context—not just the positive effects.

Thus, of all the variables analyzed, only the inefficiency score and mental quality of life (QoLM) showed significant differences between occupational groups in our sample. In this case, too, all variables of interest were completed for all participants; no missing data were recorded.

### 3.5. Correlations Between Burnout and Body Composition

#### 3.5.1. Total Muscle Mass

Muscle mass showed significant positive correlations with exhaustion (*p* = 0.006, r = 0.280) and total burnout (*p* = 0.016, r = 0.247).

#### 3.5.2. Bone Mass

Bone mass was positively correlated with exhaustion (*p* = 0.018, r = 0.243) and total burnout (*p* = 0.033, r = 0.219), such that participants with high burnout scores were significantly more likely to have high bone mass scores and vice versa (Table 4).

#### 3.5.3. Basal Metabolic Rate

The basal metabolic rate showed significant positive correlations with exhaustion (*p* = 0.025, r = 0.231) and total burnout (*p* = 0.033, r = 0.219), such that participants with high burnout scores were significantly more likely to have high basal metabolic rates and vice versa (Table 4).

#### 3.5.4. Mental Quality of Life (QoLM)

Mental quality of life was significantly negatively correlated with burnout scores (*p* < 0.05), indicating that higher burnout levels were associated with lower mental quality of life.

#### 3.5.5. Non-Significant Associations

No significant correlations were found between burnout scores and BMI, subcutaneous or visceral adipose tissue, water content, metabolic age, muscle strength, or physical quality of life (all *p* ≥ 0.05) (Table 4).

Therefore, from the correlational analysis performed, only muscle mass, bone mass, basal metabolism, and QoLM showed statistically relevant associations with the burnout scores.

These analyses are based on unadjusted group comparisons and bivariate correlations. Given the heterogeneity of age, sex, and occupational category in our sample, these results reflect exploratory associations rather than independent relationships.

## 4. Discussion

This study highlights significant associations between burnout, body composition parameters, and quality of life among medical staff, with notable differences across occupational categories. In particular, the mental quality of life and selected metabolic parameters (muscle mass, bone mass, basal metabolism) were associated with burnout dimensions.

Burnout syndrome in medical staff remains a significant challenge, not only in terms of differential diagnosis, overlapping with conditions such as post-traumatic stress, depression, or anxiety, but also regarding its physiological correlates. Increasing attention has been directed toward identifying objective markers of burnout, including biochemical, endocrine, and metabolic indicators, which may contribute to defining a potential “burnout phenotype” [19].

A previous study identified changes in inflammatory and metabolic markers in patients with burnout compared to the control group. The reported markers included hsCRP, TNF-α, IL-6, fibrinogen, D-dimers, PAI-1, HbA1c, lipid changes (HDL, TC/HDL ratio), DHEA-S, blood pressure, waist-to-hip ratio, and body fat percentage [19].

In contrast, the present study explored these associations indirectly through bioimpedance-derived measures, including muscle mass, adipose tissue, hydration status, bone mass, visceral fat, metabolic age, and basal metabolism; these parameters were correlated with burnout scores and dimensions of physical and mental quality of life.

Importantly, the present findings are correlational and descriptive. While we discuss possible physiological or metabolic explanations for observed associations, these remain hypotheses and are not directly measured in this study. The focus of our results is on occupational patterns and associations between burnout and body composition, rather than direct evidence of biological stress responses.

In addition, while we observe associations between burnout scores and body composition parameters, these findings are descriptive and correlational. Potential confounding factors such as age, sex, and occupational category may influence the results, and causal inferences cannot be drawn from the current study design.

Occupational differences were evident, with resident physicians showing higher muscle mass, bone mass, basal metabolism, and inefficiency scores compared to specialist/primary physicians, with statistically significant differences. Resident physicians also had significantly lower values for subcutaneous and visceral adipose tissue, metabolic age, and mental quality of life (QoLM) and significantly higher water levels compared to nurses and nursing assistants.

These findings may be partly explained by the demographic structure of the groups, particularly sex and age distribution. The group of resident physicians was predominantly male, while the group of specialist/primary care physicians was predominantly female.

Importantly, chronological age itself is a key determinant of metabolic characteristics; chronological age directly affects metabolism: as age increases, metabolic rate and body composition change, and metabolic age is influenced by age, sex, physical activity, and other sociodemographic factors [20].

Moreover, physiological evidence indicates that age is associated with changes in muscle mass and metabolism, contributing to differences in resting energy expenditure and body composition. While there is an observed increase in basal metabolism in younger and more active individuals, these effects may interact with age, occupation-related activity levels, and body composition [21].

Data from the literature further suggest that factors such as thermogenesis and sustained physical activity modulate basal metabolism, while advancing age is associated with identifiable shifts in the metabolic rate independent of occupation [22].

The predominance of young resident doctors in our sample may also contribute to the observed results. Occupational factors such as workload, extended working hours, and irregular nutrition and hydration may negatively influence quality of life, particularly physical components. With regard to mental quality of life, these findings may suggest a potential role of psychological resilience associated with younger age, despite limited experience and increased professional demands [22].

A significant portion of the resident sub-group had a surgical specialty and participated in prolonged surgical procedures, requiring the use of lead-based suits during investigations requiring radiation and tolerance to low temperatures. In addition to these procedural demands, resident physicians are also exposed to sustained occupational strain generated by institutional workflows, frequent interdepartmental movement, and the cumulative physical demands in the daily clinical responsibilities. All of these elements together with younger age, may contribute to the increased basal metabolic rates observed in this group [22].

However, in terms of the total burnout score, cynicism, and exhaustion among resident physicians, our results were not statistically significant. When compared to specialist/primary physicians, the inefficiency scores were significantly higher. Previous studies report heterogeneous findings, with some indicating improved efficiency in structured training environments, while others associate younger age and limited resources with increased burnout risk [23,24].

The lower metabolic age of resident physicians is consistent with their chronological age, while also reflecting the ideal profile of the other variables (low adipose and visceral tissue, high hydration level). The data in the literature remain limited regarding the role of metabolic age in determining burnout. Some results refer to the importance of subjective age, i.e., the age at which an individual perceives themselves and which is not synonymous with either chronological age or metabolic age but is nevertheless a link between the two. Thus, according to this study, older people who have an advanced subjective age are also associated with emotional exhaustion and a decline in mental quality of life, while young people who consider themselves to have an advanced subjective age are, in fact, signs of an increased mental quality of life and maturity [25].

A recent study conducted on a group of nurses showed that the level of exhaustion varied proportionally with chronological age and also with the level of physical and mental activity. These data indirectly support our findings, suggesting the implications of occupation, chronological age, and intrinsic metabolic age in predicting the onset of burnout [26].

At the same time, emotional exhaustion has been associated with increased levels of cholesterol and triglycerides, requiring the assessment of coronary risk in those with burnout [27]. It is assumed that the release of corticosteroids and catecholamines due to stress induces acute phase reactants that stimulate insulin resistance and predispose to the development of type I diabetes. On the other hand, obesity appears to be involved in reducing the rate of exhaustion [28,29].

Burnout among healthcare staff is important because it can affect the quality of medical care and increase the risk of errors. Stress reduction interventions—from relaxation techniques or cognitive-behavioral therapy to reflective groups (e.g., Balint Group-type groups)—have been proposed as strategies for preventing and decreasing burnout. In some reports, these methods have reduced emotional exhaustion and improved psychological well-being, but the evidence is still insufficient to support that prevention or improvement is sustained in the long term [30,31,32,33,34,35].

To achieve real and lasting protection against burnout, integrated approaches are needed that combine individual interventions with organizational changes—i.e., psychosocial support, restructuring of the work environment, and institutional policies to reduce occupational stress. Most intervention programs proposed to date include psychosocial and institutional support and team training [36].

In line with our results, mental quality of life, unlike physical quality of life, influences the occurrence of burnout syndrome and its subsidiary dimensions (exhaustion, cynicism, inefficiency). In a study conducted on a group of doctors in China, resilience interventions were proposed both to increase mental quality of life and to combat burnout syndrome among them [37].

Similarly, another study conducted on a group of emergency doctors in Pakistan demonstrated a strong bidirectional correlation between their level of burnout and their mental quality of life. These data confirm our findings that there is a bidirectional relationship between burnout syndrome with its three dimensions and the mental component of quality of life [38].

A retrospective study of the COVID-19 pandemic demonstrated the persistence of psychological stress symptoms and the impairment of mental quality of life in a sample of medical personnel in Italy [39]. Supporting the mental health of healthcare workers appears to be an essential part of the public health response to the COVID-19 pandemic. Recent recommendations for optimizing the professional well-being of healthcare workers include measures to support mental quality of life. For example, as proposed in previous studies, it seems important to promote compassion satisfaction among these healthcare staff by adopting strategies to adequately manage sensitivity and empathy and to compensate for exhaustion and adverse psychological effects [40,41,42,43,44].

The bioimpedance analysis in our cohort revealed higher burnout scores in participants with increased muscle mass, bone mass, and basal metabolism. Recent research indicates that occupational stress and burnout are associated with distinct alterations in metabolic biomarkers in healthcare staff, suggesting that chronic work-related stress can be reflected in metabolic profiles [45]. Evidence also shows that fat-free mass, including muscle mass, is a primary determinant of resting energy expenditure, supporting our findings that individuals with higher lean mass exhibit higher basal metabolism [46]. Additionally, studies demonstrate a direct correlation between muscle mass and resting metabolic rate after accounting for age and sex, indicating that differences in body composition may support the metabolic variations observed in burnout [47].

In a group of athletes, the relationship between ferritin deficiency and a vitamin D metabolite, 25-OH vitamin D, and the level of exhaustion was evaluated. Previously, an inverse association between these biochemical elements and depression had been found. The results were similar for exhaustion scores, indicating that male participants with vitamin D deficiency had lower muscle and bone mass and higher exhaustion scores. In contrast, in female participants, low ferritin levels were associated with the same consequences [46].

The previous results and ours suggest benefits in continuing research on the impact of burnout on body composition, highlighting the importance of associations with burnout alongside psychological and physical outcomes. Our data also suggest that being overweight or obese and having a high percentage of subcutaneous adipose tissue mainly affects the physical quality of life and not the mental quality of life. We explain these results in the context of relatively preserved psychological resilience, despite the absence of proper nutrition and, most likely, adequate physical activity. The latter is undoubtedly very important, as it has multiple beneficial effects on physical, mental, and spiritual health and well-being. Physical exercise includes any movement or muscular activity that results in calorie loss [47,48,49,50,51,52].

This reality is particularly relevant for healthcare staff: rotations, shifts, demanding tasks, and variable professional uncertainty make it difficult to plan and carry out regular physical exercise. As a result, healthcare staff are at increased risk of compromising their physical and mental quality of life, which can exacerbate the susceptibility to exhaustion and burnout [53,54].

Our analysis indicates that among the parameters measured, muscle mass, bone mass, and basal metabolism are significantly associated with burnout scores and mental quality of life. This suggests that physiological or lifestyle-related changes may represent an important—but not exclusive—component associated with burnout, complementary to the psychological components of burnout.

However, not all variables showed significant associations, underscoring the need for careful selection of relevant body composition parameters in future research.

Data from the literature support the existence of metabolic and inflammatory changes in individuals exposed to chronic stress, although the findings remain inconsistent. This variability suggests that the relationship between burnout and physiological parameters is complex and likely multifactorial [20,21].

Importantly, all observed associations in this study are correlational and do not provide direct evidence of underlying biological mechanisms. Future longitudinal and mechanistic studies are needed to clarify these relationships.

### Limitations Recommendations, and Directions for Future Research

This study has several limitations. The modest sample size and uneven distribution across sex, age, and occupation may reduce the statistical power and increase the risk of type II errors. The cross-sectional design precludes causal inference, and unmeasured confounders—such as lifestyle, work schedule, occupational stress, seasonal factors, and medical specialty—may have influenced both the body composition and burnout scores. Finally, the limited variables assessed, without inflammatory, hormonal, immunological, or genetic biomarkers, restrict the exploration of mechanistic associations.

Future studies should address these limitations through longitudinal designs with repeated body composition measurements, inclusion of biological markers (inflammatory, hormonal, immunological, genetic), and rigorous control of confounders to clarify the independence of observed associations. Comparisons of BIA with reference methods (e.g., DXA), supplemented by biochemical analyses, are recommended to improve the accuracy. Interventional studies targeting body composition modification (diet, exercise, hydration) and evaluating effects on burnout and quality of life are warranted to explore causal relationships [55,56].

## 5. Conclusions

The tangentiality of stress to all mechanisms maintaining the body’s homeostasis is controversial, and identifying them means guaranteeing a targeted plan to prevent and combat burnout syndrome. This study provides preliminary and exploratory evidence of associations between burnout and body composition, highlighting molecular aspects that remain largely unexplored. Significant occupational differences were observed: resident physicians exhibited higher muscle and bone mass, basal metabolism, and inefficiency scores, alongside lower adipose tissue, metabolic age, and mental quality of life compared to specialist/primary physicians. Given the cross-sectional design, small sample size, and absence of multivariate adjustment for potential confounders, these findings represent correlational patterns rather than evidence of causality. Methodological limitations, particularly the use of BIA, further caution against overgeneralization to other healthcare populations or settings. Nonetheless, BIA offers rapid accessible preliminary insights into body composition patterns associated with burnout, which may guide future research, preventive interventions, and longitudinal studies using more robust physiological markers.

## Figures and Tables

**Table 1:** Characteristics of the sample analyzed in the study.

Parameter	Value
**Occupation (No., %)**	
Nursing assistants	15 (15.8%)
Nurse	31 (32.6%)
Resident physician	36 (37.9%)
Specialist/primary physician	13 (13.7%)
**Department (No., %)**	
Medical	72 (75.8%)
Surgical	23 (24.2%)
**Age**	
Mean ± SD	39.93 ± 10.29
Median (IQR)	38 (31–49)
**Age category (No., %)**	
≤35 years	40 (42.1%)
>35 years	55 (57.9%)
**Sex (No., %)**	
Female	72 (75.8%)
Male	23 (24.2%)

**Table 2:** The relationship between body composition and occupation.

	Total (N = 95)		Specialist and Primary Care Physicians (N = 13)		Resident Physicians (N = 36)		Nurses (N = 31)		Nursing Assistants (N = 15)		*p* *
	Mean ± SD	Median	Mean ± SD	Median	Mean ± SD	Median	Mean ± SD	Median	Mean ± SD	Median	
BMI	26.38 ± 5.48	25.12 (22.05–29.87)	23.43 ± 4.12	23 (20.5–25)	25.54 ± 4.82	23.9 (21.8–28.2)	27.27± 5.32	25.9 (22.5–31.9)	29.11 ± 7	27.7 (23.4–34.4)	**0.048**
Muscular mass	48.20 ± 10.47	45.36 (40.47–43.03)	43.74 ± 9.46	40.3 (38.5–48.5)	52.6 ± 12.43	52.55 (41.2–60.8)	45.59 ± 7.25	44.1 (40.6–48.7)	46.91 ± 8.66	44.5 (41.6–50.5)	**0.033**
Subcutaneous adipose tissue	30.65 ± 10.31	31.55 (24.58–39.12)	31.3 ± 10.28	31.5 (24.3–36.9)	25 ± 9.54	26 (18.33–31.5)	35 ± 8.13	34.5 (28.5–43.8)	34.66 ± 10.6	34.2 (27.2–44.3)	**<0.001**
Water content	50.19 ± 6.86	49.3 (45.3–54.01)	50.72 ± 6.32	50 (47.2–55.05)	53.75 ± 6.77	54 (49.1–59.7)	47.64 ± 5.77	47.3 (42.4–52.3)	46.46 ± 5.8	45.9 (42.5–49)	**<0.001**
Bone mass	2.57 ± 0.60	2.4 (2.08–2.82)	2.4 ± 0.8	2.1 (1.95–2.6)	2.78 ± 0.62	2.8 (2.2–3.18)	2.43 ± 0.49	2.4 (2.1–2.7)	2.48 ± 0.45	2.3 (2.1–2.8)	**0.025**
Visceral adipose tissue	6.16 ± 3.74	5.68 (3.65–8.37)	4.6 ± 3.2	3.5 (2.5–5.5)	5.1 ± 3.74	4.25 (2.13–8)	7.32 ± 3.71	7 (5–11)	7.63 ± 3.19	8 (5–9)	**0.005**
Metabolic age	40.95 ± 17.09	42.12 (31.3–53.3)	36.85 ± 17.4	34 (24–46.5)	32 ± 14.8	32.5 (19.2–42.7)	48.77 ± 15.5	46 (38–64)	49.8 ± 14.11	56 (44–60)	**<0.001**
Muscle strength	3.72 ± 1.59	3.75 (2.25–5)	3.46 ± 1.61	4 (2–5)	4.08 ± 1.55	5 (3–5)	3.52 ± 1.71	3 (2–5)	3.47 ± 1.35	3 (2–5)	0.336
Basal metabolism	1535.6 ± 321.2	1766.97 (1268.3–1658.8)	1342.4 ± 278.9	1262 (1167–1454)	1666.3 ± 374.8	1636.5 (1337.2–1857.2)	1485.8 ± 227.4	1428 (1300–1576)	1492.4 ± 279.6	1399 (1269–1748)	**0.005**

* Kruskal–Wallis H test, BMI: Body Mass Index.

**Table 3:** Exhaustion, cynicism, inefficiency, burnout, QoLF, and QoLM by occupation.

	Total		Specialist and Primary Care Physicians		Resident Physicians		Nurses		Nursing Assistants		*p* *
	Mean ± SD	Median	Mean ± SD	Median	Mean ± SD	Median	Mean ± SD	Median	Mean ± SD	Median	
Exhaustion	12.5 ± 7.28	11.75 (6.12–19.25)	12.4 ± 7.88	8 (6.5–19)	14.7 ± 6.47	16 (9–20)	11.3 ± 6.9	11 (5–17)	11.6 ± 7.88	12 (4–21)	0.220
Cynicism	7.61 ± 6.6	6.5 (2.18–12.25)	8.62 ± 7.68	7 (1.5–15)	8.69 ± 6.64	8 (3.25–12)	5.81 ± 5.98	5 (2–9)	7.33 ± 6.12	6 (2–13)	0.276
Inefficiency	6.95 ± 4.84	4.87 (1.12–11)	7 ± 8.01	4 (0.5–13)	8.36 ± 5.81	6.5 (4–13)	4.45 ± 5.34	3 (0–7)	8 ± 8.23	6 (0–11)	**0.049**
Total burnout	27.11 ± 18.32	23.25 (12.43–40.93)	28.23 ± 22.9	20 (9–44.5)	31.75 ± 15.9	32 (18.75–47.25)	21.55 ± 15.4	19 (9–26)	26.93 ± 19.1	22 (13–46)	0.064
QoLF	48.64 ± 7.41	49.23 (43.75–55.05)	51.3 ± 6.36	52.45 (48.7–56.9)	50.1 ± 6.87	51.16 (44.4–54.9)	46.76 ± 7.2	47.95 (40.6–53.5)	46.4 ± 9.21	45.36 (41.3–54.9)	0.093
QoLM	45.45 ± 8.13	45.45 (38.85–52.67)	41.43 ± 8.36	40.5 (34.5–49.2)	42.84 ± 9.96	44.8 (34.7–51.3)	48.15 ± 8.15	47.8 (41.5–55.8)	49.94 ± 6.07	48.7 (44.7–54.4)	**0.006**

* Kruskal–Wallis H test. QoLF: Quality of physical life, QoLM: Quality of mental life.

**Table 4:** Correlation between burnout scores and the parameters studied.

	Total Burnout		Exhaustion		Cynicism		Inefficiency	
	*p* *	r	*p* *	r	*p* *	r	*p* *	r
BMI	0.693	0.041	0.411	0.085	0.838	−0.021	0.994	−0.001
Muscular mass	**0.016**	**0.247**	**0.006**	**0.280**	0.136	0.154	0.226	0.125
Subcutaneous adipose tissue	0.211	−0.130	0.575	−0.058	0.136	−0.154	0.401	−0.087
Water content	0.294	0.109	0.627	0.051	0.156	0.147	0.479	0.073
Bone mass	**0.033**	**0.219**	**0.018**	**0.243**	0.165	0.143	0.284	0.111
Visceral adipose tissue	0.990	0.001	0.479	0.073	0.531	−0.065	0.614	−0.052
Metabolic age	0.492	−0.071	0.994	0.001	0.259	−0.117	0.545	−0.063
Muscle strength	0.611	0.053	0.639	0.049	0.650	−0.047	0.874	0.016
Basal metabolism	**0.033**	**0.219**	**0.025**	**0.231**	0.153	0.148	0.271	0.114
QoLF	0.426	−0.083	0.248	−0.120	0.751	−0.033	0.720	−0.037
QoLM	**0.002**	**−0.315**	**0.002**	**−0.308**	**0.002**	**−0.313**	**0.010**	**−0.264**

* Spearman’s rho correlation coefficient. BMI: Body Mass Index, QoLF: Quality of physical life, QoLM: Quality of mental life.

## Data Availability

The data that support the findings of this study are available from the corresponding author upon reasonable request, due to restrictions related to participant privacy and ethical considerations.
